# Proteomic analysis of *Clostridium thermocellum* core metabolism: relative protein expression profiles and growth phase-dependent changes in protein expression

**DOI:** 10.1186/1471-2180-12-214

**Published:** 2012-09-21

**Authors:** Thomas Rydzak, Peter D McQueen, Oleg V Krokhin, Vic Spicer, Peyman Ezzati, Ravi C Dwivedi, Dmitry Shamshurin, David B Levin, John A Wilkins, Richard Sparling

**Affiliations:** 1 Department of Microbiology, University of Manitoba, Winnipeg, MB, R3T 2N2, Canada; 2 Department of Internal Medicine & Manitoba Centre for Proteomics and Systems Biology, University of Manitoba, Winnipeg, MB, R3E 3P4, Canada; 3 Department of Physics & Astronomy, University of Manitoba, Winnipeg, MB, R3T 2N2, Canada; 4 Department of Biosystems Engineering, University of Manitoba, Winnipeg, MB, R3T 3V6, Canada

## Abstract

**Background:**

*Clostridium thermocellum* produces H_2_ and ethanol, as well as CO_2_, acetate, formate, and lactate, directly from cellulosic biomass. It is therefore an attractive model for biofuel production via consolidated bioprocessing. Optimization of end-product yields and titres is crucial for making biofuel production economically feasible. Relative protein expression profiles may provide targets for metabolic engineering, while understanding changes in protein expression and metabolism in response to carbon limitation, pH, and growth phase may aid in reactor optimization. We performed shotgun 2D-HPLC-MS/MS on closed-batch cellobiose-grown exponential phase *C. thermocellum* cell-free extracts to determine relative protein expression profiles of core metabolic proteins involved carbohydrate utilization, energy conservation, and end-product synthesis. iTRAQ (isobaric tag for relative and absolute quantitation) based protein quantitation was used to determine changes in core metabolic proteins in response to growth phase.

**Results:**

Relative abundance profiles revealed differential levels of putative enzymes capable of catalyzing parallel pathways. The majority of proteins involved in pyruvate catabolism and end-product synthesis were detected with high abundance, with the exception of aldehyde dehydrogenase, ferredoxin-dependent Ech-type [NiFe]-hydrogenase, and RNF-type NADH:ferredoxin oxidoreductase. Using 4-plex 2D-HPLC-MS/MS, 24% of the 144 core metabolism proteins detected demonstrated moderate changes in expression during transition from exponential to stationary phase. Notably, proteins involved in pyruvate synthesis decreased in stationary phase, whereas proteins involved in glycogen metabolism, pyruvate catabolism, and end-product synthesis increased in stationary phase. Several proteins that may directly dictate end-product synthesis patterns, including pyruvate:ferredoxin oxidoreductases, alcohol dehydrogenases, and a putative bifurcating hydrogenase, demonstrated differential expression during transition from exponential to stationary phase.

**Conclusions:**

Relative expression profiles demonstrate which proteins are likely utilized in carbohydrate utilization and end-product synthesis and suggest that H_2_ synthesis occurs via bifurcating hydrogenases while ethanol synthesis is predominantly catalyzed by a bifunctional aldehyde/alcohol dehydrogenase. Differences in expression profiles of core metabolic proteins in response to growth phase may dictate carbon and electron flux towards energy storage compounds and end-products. Combined knowledge of relative protein expression levels and their changes in response to physiological conditions may aid in targeted metabolic engineering strategies and optimization of fermentation conditions for improvement of biofuels production.

## Background

*Clostridium thermocellum* ATCC 27405, an anaerobic, Gram-positive thermophilic bacterium, is capable of cellulosome-mediated breakdown of (hemi)cellulose [1,2] and simultaneous fermentation of resulting cello-oligosaccharides into hydrogen (H_2_) and ethanol [3-5]. This reduces the need for separate cellulase production, cellulose hydrolysis, and fermentation, which could improve economic viability of industrial cellulosic biofuel production [4,6,7]. Among cellulolytic microorganisms, *C. thermocellum* exhibits one of the highest growth rates on cellulose [8-10]. Its high temperature growth optimum aids in H_2_ recovery [11], and the availability of annotated genome sequence (GenBank accession number ZP_00312459.1) allows for deduction of metabolic pathways *in silico*, expression studies by microarray and proteomic analysis, and genetic engineering [12-14]. It is therefore an attractive model for biofuel production via consolidated bioprocesing.

Despite these appealing characteristics, *C. thermocellum* normally produces both ethanol and H_2_ with yields (~0.6 and 1.3 mol per mol hexose, respectively) well below the ‘Thauer limit’ of either 2 moles of ethanol or 4 moles of H_2_ per mole hexose, respectively [4,7]. This is due to branched fermentative pathways that lead to the production of both ethanol and H_2_ (with concomitant production of CO_2_ and acetate), as well as branches leading to formic acid and lactic acid that compete for carbon and/or electrons required for the production of either ethanol or H_2_[4,6,7]. Metabolic engineering strategies to improve product yields in *C. thermocellum*[15] and related species [16] have been only moderately successful and at times resulted in unpredicted changes in product yields [12]. This may be due to the complexity of metabolic networks in which multiple gene products may catalyze parallel reactions [4], the presence of response regulators that modulate gene and gene-product expression [17-19], and modulation of enzyme activity via intracellular metabolite levels [20,21]. While many of the genes and proteins involved in pyruvate catabolism and product formation have been verified via RT-PCR [22], enzyme activity assays [4], and purification [23,24], a more thorough understanding of metabolic and regulatory networks must be attained.

A number of studies have demonstrated the ability of *C. thermocellum* to control scaffoldin and cellulase mRNA [25-28] and protein [29-32] levels in response to substrate type and growth rate, whereby cellulosome gene expression is positively regulated through binding of cellulose and xylan to anti-σ factors, preventing their binding to alternative σ factors required for cellulosome expression [33,34], and negatively regulated by cellobiose via a carbon catabolite repression mechanism [28,31]. A few studies have looked at expression levels of genes encoding proteins involved in central metabolism and end-product formation. Stevenson and Weimer have looked at expression levels of 17 genes involved in cellulose degradation, intracellular phosphorylation, catabolite repression, and fermentation end-product formation in response to substrate and growth rate [35]. More recently, microarray studies have looked at overall gene expression levels and global changes in mRNA levels in response to substrate and dilution rate [36] and growth phase in cellulose-grown batch cultures [37]. To date, there have been no reports of global protein expression levels of *C. thermocellum*.

We have now completed the first proteomic study of cellobiose-grown batch culture *C. thermocellum* cell-free extracts to determine relative abundances of metabolic proteins and responses in their expression levels during different growth phases. Shotgun two-dimensional high performance liquid chromatography-tandem mass spectrometry (2D-HPLC-MS/MS) was used to determine protein relative abundance indexes (RAI), calculated as the number of spectral counts (SpC) divided by molecular mass (Mr) of protein, in exponential phase cell-free extracts. Differences in protein expression levels between exponential and stationary phase cell-free extracts labeled with isobaric tags for relative and absolute quantitation (iTRAQ) were determined using 4-plex 2D-HPLC-MS/MS.

## Materials and methods

### Organism, media, and growth

The type strain of *Clostridium thermocellum*, DSM 1237 (equivalent to ATCC 27405), obtained from the German Type Culture collection, was employed for all growth experiments. Fresh cultures were maintained by routinely transferring 10% (v/v) mid-exponential phase inoculum into complex 1191 medium as previously described [4] containing 2.2 g L^-1^ (11.8 mM) cellobiose. Cultures were grown at 60°C and stored anaerobically at 4°C. All chemicals were reagent grade and were obtained from Sigma Chemical Co (St. Loius, MO) unless otherwise specified. All gases were purchased from Welder’s Supply (Winnipeg, MB, Canada).

Cells for end-product and proteomic analysis were grown in triplicate in anaerobic Balch tubes (26 mL; Bellco Glass Inc., Vineland, NJ) in 10 mL of 1191 medium (pH 7.2) on 2.2 g L^-1^ cellobiose. Media preparation and inoculation protocols were followed as described by Islam *et al.*[3]. Samples for end-product, cell biomass, and pH measurements were taken throughout growth, while samples for proteomic analysis were taken in exponential and stationary phase (OD_600_ ~ 0.37 and ~0.80, respectively).

### Cell growth, pH, and end-product analysis

Cell growth was monitored spectrophotometrically (Biochrom, Novaspec II) at 600 nm. Sample processing, pH measurement, product gas, protein, sugar, and end-product analyses were performed as previously described [4]. Data are presented as the means of three biological replicates. Elemental biomass composition (in mM) was calculated from protein content using a molecular weight of 101 g mol^-1^, corresponding to the average composition of cell material (C_4_H_7_O_2_N) based on a stoichiometric conversion of cellobiose into cell material [38]. Barometric pressure, test tube pressure, and gas solubility in water were taken into account during calculation of gas measurements [39]. Bicarbonate equilibrium was taken into account for CO_2_ quantitation [40].

### Preparation of cell-free extracts for proteomic analysis

Exponential and stationary phase cell cultures (10.5 mL) were centrifuged (10000 × g, 5 minutes, 4°C). Cells pellets were washed 3 times in 500 μL 1x PBS buffer and then frozen at −80°C. Cell pellets were re-suspended in 540 μL lysis buffer (Tris–HCl, 10 mM, pH 7.4; CaCl_2_, 3 mM; 2 mM MgCl_2_, 2 mM; bacterial protease inhibitor, 1.0%; Tergitol NP-40, 0.1%) and sonicated 5 rounds for 15 seconds each round with cooling on ice in between rounds. Unlysed cells were removed by centrifugation (14000 × g, 10 minutes) and protein concentration of supernatant was determined Bicinchononic Acid (BCA) Protein Assay Kit (Pierce Biotechnology, Rockford, IL) as outlined by the manufacturer. Supernatant was stored at −80°C. An aliquot corresponding to 200 μg of protein was mixed with 100 mM ammonium bicarbonate, reduced with dithiothreitol (10 mM), and incubated for 30 minutes at 57°C. Proteins were then alkylated with iodoacetamide (50 mM) for 30 minutes at room temperature in the dark. Excess iodoacetamide was quenched with dithiothreitol (16 mM). Peptides were digested in a 1:50 trypsin/protein ratio (Promega, Madison, WI) for 10 hours at 37°C. Samples were then acidified with an equal volume of 3% trifluoroacetic acid (TFA), lyophilized, and re-suspended in 270 μL of 0.1% TFA. Samples were loaded on a C18 X-Terra column (1 × 100 mm, 5 μm, 100 Å; Waters Corporation, Milford, MA, USA), desalted using 0.1% TFA, and peptides were eluted with 50% acetonitrile. Desalted samples were stored at −80°C for 2D-HPLC-MS/MS analysis. For comparative proteomic analysis of exponential and stationary phase cells, each trypsinized protein sample (100 μg) was labelled with isobaric Tags for Relative and Absolute Quantitation (iTRAQ) reagent (Applied Biosystems, Foster City, CA, USA) as outlined by the manufacturer. Samples differentially labelled with isobaric tags of different masses (exponential phase replicate A [iTRAQ tag 114], exponential phase replicate B [iTRAQ tag 115], stationary phase replicate A [iTRAQ tag 116], stationary phase replicate B [iTRAQ tag 117]) were mixed in equal proportions and subjected to 2D-HPLC-MS/MS [41,42].

### Two-dimensional high-performance liquid chromatography-mass spectrometry analysis

Trypsinized peptides with or without iTRAQ label were separated in the first dimension using an Agilent 1100 Series HPLC system (Agilent Technologies, Wilmington, DE). Samples were injected onto a C18 X-Terra column (1 × 100 mm, 5 μm, 100 Å; Waters Corporation, Milford, MA, USA) and eluted with a linear water-acetonitrile gradient (20 mM ammonium formate, pH 10, in both eluents A and B, 1% acetonitrile/min, 150 μL/min flow rate). A concentrated 200 mM solution of ammonium formate at pH 10 was prepared as described by Gilar *et al.*[43]. Buffers A and B for first-dimension separation were prepared by a 1/10 dilution of this concentrated buffer with water and acetonitrile, respectively. Fifty 1-min fractions were collected (roughly 6.6 μg/fraction). Samples were concatenated (fraction 1 and 31, 2 and 32, etc.) into a total of 25 fractions as described by Dwivedi *et al.* [44]. Each was lyophilized and re-suspended in 100 μL of 0.1% formic acid. A splitless nanoflow Tempo LC system (Eksigent, Dublin, CA, USA) with 20 μL sample injection via a 300 μm × 5 mm PepMap100 precolumn and a 100 μm × 150 mm analytical column packed with 5 μm Luna C18(2) (Phenomenex, Torrance, CA) was used in the second-dimension separation prior to tandem MS analysis. Both eluents A (2% acetonitrile in water) and B (98% acetonitrile) contained 0.1% formic acid as ion-pairing modifier. A 0.33% acetonitrile/min linear gradient (0-30% B) was used for peptide elution, providing a total 2 hour run time per fraction in the second dimension.

### Mass spectrometry

A QStar Elite mass spectrometer (Applied Biosystems, Foster City, CA) was used in standard MS/MS data-dependent acquisition mode with a nano-electrospray ionization source. The 1 s survey MS spectra were collected (*m*/*z* 400*–*1500) followed by three MS/MS measurements on the most intense parent ions (80 counts/s threshold, +2 to +4 charge state, *m*/*z* 100–1500 mass range for MS/MS), using the manufacturer’s “smart exit” settings and iTRAQ settings. Previously targeted parent ions were excluded from repetitive MS/MS acquisition for 60 s (50 mDa mass tolerance).

### Database search, protein identification, and statistical analysis

Raw spectra WIFF files of unlabeled peptides were treated using standard script (Analyst QS 2.0) to generate text files in Mascot Generic File format (MGF) [45] and ProteoWizard to generate mzML files [46]. MGF files containing the MS/MS spectra information for all 25 fractions were concatenated and submitted for protein identification using Global Proteom Machine’s (GPM) X!Tandem [47] and an in-house GPU-based peptide identification engine described by McQueen *et al.*[48]. Standard QTOF settings were used for the search: 100 ppm and 0.4 Da mass tolerance for parent and fragment ions, respectively. Permitted amino acid modifications included constant carbamidomethylation of Cys. All mass spectrometry data, including MS/MS MGF files and corresponding XML files containing peptide and protein identifications, is archived in the Manitoba Centre for Proteomics and Systems Biology GPM server ( http://140.193.59.2). The accession numbers (‘lookup model’) for the shotgun 2D-HPLC-MS/MS run and iTRAQ 4-plex 2D-HPLC-MS/MS run are 01700007037 and 02M00007915, respectively. The “relative abundance index” (RAI) for each protein was calculated as the number of spectral counts (SpC) divided by molecular mass (Mr) of protein.

Spectra files of iTRAQ labelled peptides were also analyzed using ProteinPilot software version 2.0.1 (Applied Biosystems/MDS Sciex, Concord, ON, Canada) using the Paragon algorithm [49]. The search parameters were complete modifications of Cys alkylation with iodoacetic acid, and inbuilt iTRAQ analysis residue modifications settings were on. The reporter ion (iTRAQ tag) intensities for each tryptic peptide identified (with expectation values < −1.5) were histogrammed by the log2 of the ratios (Z0 = tag_116_/tag_114_, Z1 = tag_117_/tag_115_, Z2 = tag_115_/tag_114_, and Z3 = tag_117_/tag_116_) to build overall peptide population distributions, where exponential phase replicates were labelled with tags 114 and 115, respectively, and stationary phase replicates were labeled with tags 116 and 117, respectively. Peptide level Z-scores are mapped as the distance from the population mean in units of standard deviation; initial protein-level Z-scores are average of the member peptide Z-score values. The Z-scores (Z2,Z3) contain information about the stability across biological replicates at the same growth state. We have devised a simple algorithm to combine these with the differential data in (Z0,Z1), expressed as the difference between the magnitudes of vectors from the origin to points (Z0,Z1) and (Z2,Z3), scaled by the widths of their peptide histogram distributions. The sign of the transformed value is determined by the angle subtended by a vector from the origin to the point (Z0,Z1). We denote this combined value as the vector difference (*V*_*diff*_). Z-scores were converted into fold-changes by taking 2 to the power of the Z-score.

## Results and discussion

### Growth and end-product synthesis

**Figure 1:**
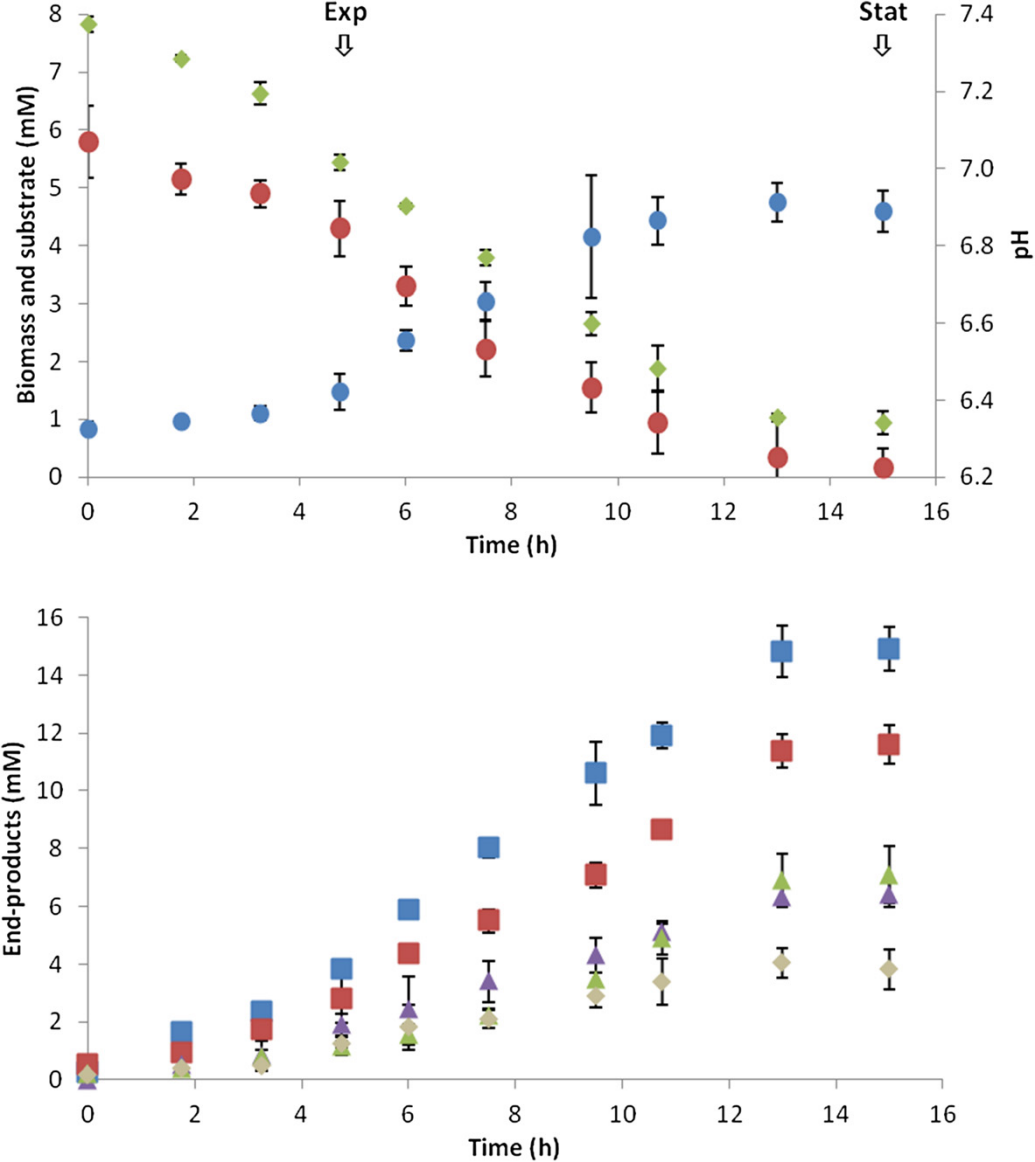
** Fermentation growth and metabolite production.** Cellobiose utilization, biomass production, pH change, and metabolite production plots of *C. thermocellum* grown in 1191 medium batch cultures on 2 g l^-1^ cellobiose. Arrows indicate sampling points for exponential and stationary phase proteomic analysis. Biomass (blue circle), cellobiose (red circle), pH (olive green diamond), H_2_ (blue square), CO_2_ (red square), acetate (purple triangle), ethanol (olive green triangle), formate (tan diamond).

In this study, we investigated the relative abundance profiles (RAI) of core metabolic proteins in exponential phase cultures, and changes in protein expression in response to growth phase. All *C. thermocellum* DSM 1237 cultures were grown in complex 1191 medium closed-batch cultures with no pH control, on 2.2 g L^-1^ cellobiose. Cell growth (as indicated by biomass production), substrate consumption, change in pH, and end-product formation during growth are shown in Figure 1. Cultures reached stationary phase in ~14 h upon exhaustion of cellobiose, suggesting carbon limited growth, and had a final biomass and pH of 4.8 mM and 6.3, respectively. In agreement with previous reports [3,4,9,35,50,51] H_2_, CO_2_, ethanol, and acetate were major end-products and paralleled growth and cellobiose consumption. A slight inversion of acetate-to-ethanol ratio was observed during the transition to stationary phase. This was also observed by Raman *et al.*[37] and could be stimulated by H_2_ build-up [2,19,50,52-55]. Formate was also a major end-product in agreement with Sparling *et al.*, Islam *et al.*, and Rydzak *et al.*[3-5,55]. The lack of formate detection in some *C. thermocellum* studies could be attributed to HPLC detection methods or media composition [56]. Lactate production was below detectable limits as expected under carbon-limited conditions [3]. Carbon recovery (91%) and O/R ratio (0.93) confirm that major end-products were accounted for. 

### Relative protein abundance using shotgun and 4-plex 2D-HPLC-MS/MS

Two-dimensional high-performance liquid chromatography-tandem mass spectrometry detected (with a 99.9% confidence score and minimum peptide detection threshold of 2) a total of 1575 of 3236 proteins, including 1468 proteins detected by shotgun 2D-HPLC-MS/MS in exponential phase cell-free extracts, and 1071 proteins detected by 4-plex 2D-HPLC-MS/MS of duplicate iTRAQ labelled exponential and stationary phase samples. We have currently focused strictly on core metabolic proteins that primarily dictate the majority of carbon and electron flux from cellulose and/or cellobiose to end-products. Putative proteins responsible for (i) carbohydrate hydrolysis, (ii) cellodextrin transport, (iii) glycolysis, (iv) energy storage, (v) pentose phosphate pathway, (vi) pyruvate catabolism, (vii) end-product synthesis, and (viii) energy generation and pyrophosphate metabolism are examined.

Determination of relative protein expression profiles is essential for targeted metabolic engineering strategies for strain improvement (ie. optimization of product titres, increasing growth rates, preventing product inhibition). In recent years, spectral counts obtained from shotgun proteomic approaches have been shown to be a good estimation of protein abundance [57-60]. Liu *et al.* demonstrated a linear correlation between spectral counts and relative protein abundance (R^2^ = 0.9997) over 2 orders of magnitude [57]. Quantitation via spectral counting shows strong correlation with isotopic label-based approaches [60], such as ^14^ N/^15^ N and precursor peak area intensity measurements [58]. Given that larger proteins generally give rise to a greater number of peptides following digestion, and thus a greater number spectral counts, relative protein abundance is commonly standardized to account for protein size. Rappsilber *et al.* used “protein abundance index” (PAI), which represents the number of peptides identified divided by the number of theoretically observed peptides, to quantify the relative abundance of proteins detected by MS analyses [61]. Zybailov *et al.* and Florens and Washburn used “normalized spectral abundance factor” (NSAF), which represents the number of spectral counts divided by protein length [62,63]. In this study, we have quantified 2D-HPLC-MS/MS abundance profiles based on each proteins “relative abundance index” (RAI), calculated as the number of spectral counts (SpC) divided by molecular mass (Mr) of protein.

While the number of proteins detected by shotgun 2D-HPLC-MS/MS was greater than 4-plex 2D-HPLC-MS/MS, RAI values followed a similar trend, further verifying general protein abundance using both acquisition methods ( Additional file 1). However, the RIA per a given protein was lower using the 4-plex versus shotgun acquisition method. This was expected given that the 4-plex run simultaneously measures four samples and associated labels, thus reducing available peptide acquisition time. Due to the increased sensitivity and deeper coverage, we use the RAI data of shotgun exponential phase samples when discussing relative protein expression profiles in the text.

### Changes in stationary phase protein expression levels using iTRAQ 2D-HPLC-MS/MS

Understanding cellular responses to pH change, end-product accumulation, and substrate limitation may aid in improving strain growth through targeted deregulation of factors that limit growth and production of desired end-products. Comparison of expression levels of two biologically replicated iTRAQ-labelled exponential phase and stationary phase samples (tagged with reporter ions 114 & 115 and 116 & 117, respectively) was performed using 4-plex 2D-HPLC-MS/MS. Ratios of z-score values among exponential and stationary phase biological replicates (reporter ion ratios 115/114 vs 117/116) and between exponential phase *vs* stationary phase samples (reporter ion ratio 116/114 *vs* 117/115) are plotted in Additional file 2a and 2b, respectively, to illustrate correlation between biological replicates. While Additional file 2a shows good correlation between biological replicates (perfect correlation represented by coordinates 0,0), a number of proteins have poorer correlation between replicates. To determine the statistical significance of protein expression ratios between exponential and stationary phase samples when factoring in the deviation between biological replicates, z-scores ratios for each protein were converted into vectors, and the vector difference was calculated (see Methods). Exponential phase *vs* stationary phase z-scores (*Z*_116/114_ and *Z*_117/115_) are plotted in Additional file 2b and are color coordinated based on vector difference. Vector differences greater than 2 represent proteins with the highest change in expression, while vector differences less than 0.5 represent proteins with little statistical change in expression. This calculation allowed us to eliminate values of high change between exponential and stationary phase samples when variation between replicates was higher than that of the change in exponential *vs* stationary phase samples. We propose that a vector difference of ≥ 0.5 as a confident change in expression between exponential and stationary phase proteins. Changes in protein expression levels were manually verified. Differences in protein expression between stationary and exponential phase cell-free extracts of core metabolic proteins are summarized in Table 1. A total of 166 of 252 encoded core metabolic proteins were detected using a combination of both shotgun and 4-plex acquisition methods. Twenty-four percent (24%) of proteins detected using 4-plex 2D-HPLC-MS/MS had a change in expression with a *V*_*diff*_ greater than 0.5. Nineteen percent (19%) of these proteins increased during the transition from exponential to stationary phase, while only 4% decreased in stationary phase, and 15% of these differentially expressed proteins changed by a magnitude greater than 1.

**Table 1:** Protein detection using shotgun (single-plex) and iTRAQ labelled 4-plex 2D-HPLC-MS/MS and relative changes in protein expression levels

**Core metabolic protein categories**	**Total genes**	**Proteins detected**			**Changes in protein levels (Stat/Exp)**	
		**1-Plex**	**4-Plex**	**Total**	***V***_***diff***_** ≥ 0.5**	
					**Increased**	**Decreased**
Non-catalytic cellulosomal proteins	8	5	6	7	0	0
Cellulosomal glycosidase	73	29	26	31	2	1
Non-cellulosomal glycosidases	35	17	13	19	3	0
RsgI-like σ-factors and anti-σ^I^ factors	9	3	2	3	0	0
Cello-oligosaccharide ABC transporters	14	9	8	10	2	1
Glycolysis	20	15	15	15	3	1
Pentose phosphate pathway	6	4	3	5	1	0
Energy storage	13	11	11	13	3	0
Pyruvate formation from phosphoenolpyruvate	8	8	8	8	0	2
End-product synthesis from pyruvate	49	39	38	41	12	0
Energy generation	17	14	14	14	2	1
**Total**	252	154	144	166	28	6

Core metabolic proteins were classified into functional categories. The total number of protein encoding genes in each category and the number of corresponding proteins detected are provided. The number of proteins that changed during transition from exponential to stationary phase were listed only when their vector difference (*V*_*diff*_) was greater than 0.5. Proteins detected can be viewed in Additional files 3and 4.

### Central carbohydrate metabolism

Global proteomic analysis is fundamental in verifying carbon utilization and end-product synthesis pathways. While mRNA expression profiles provide a great wealth of information with regards to transcriptional patterns, proteomics can rectify the discrepancy between transcription and translation. Relative protein expression profiles allow us to deduce which proteins, and therefore pathways, are utilized during carbohydrate metabolism. Furthermore, changes in protein levels in response to growth phase may help in hypothesizing regulatory elements that may be targeted for increasing product yields during monoculture and co-culture fermentation processes. Below we discuss key proteins involved in carbohydrate utilization and transport, glycolysis, energy storage, pentose phosphate production, pyruvate catabolism, end-product synthesis, and energy production.

### Proteins involved in cellulose and (hemi)cellulose degradation and transport

#### Cellulose hydrolysis

*C. thermocellum* encodes a number of carbohydrate active enzymes (CAZymes) allowing for efficient degradation of cellulose and associated polysaccharides (Carbohydrate Active Enzyme database; http://www.cazy.org/). These include (i) endo-β-glucanases, which cleave internal amorphous regions of the cellulose chain into shorter soluble oligosaccharides, (ii) exo-β-glucanases (cellodextrinases and cellobiohydrolases), which act in a possessive manner on reducing or nonreducing ends of the cellulose chain liberating shorter cellodextrins, and (iii) β-glucosidases (cellodextrin and cellobiose phosphorylases), which hydrolyze soluble cellodextrins ultimately into glucose [10]. Other glycosidases that allow hydrolysis of lignocellulose include xylanases, lichenases, laminarinases, β-xylosidases, β-galactosidases, and β-mannosidases, while pectin processing is accomplished via pectin lyase, polygalacturonate hydrolase, and pectin methylesterase [64,65]. These glycosidases may be secreted as free enzymes or may be assembled together into large, cell-surface anchored protein complexes (“cellulosomes”) allowing for the synergistic breakdown of cellulosic material. The cellulosome consists of a scaffoldin protein (CipA) which contains (i) a cellulose binding motifs (CBM) allowing for the binding of the scaffoldin to the cellulose fiber, (ii) nine type I cohesion domains with that mediate binding of various glycosyl hydrolases via their type I dockerin domains, and (iii) a type II dockerin domain which mediates binding to the type II cohesion domain found on the cell-surface anchoring proteins. The cell-surface anchoring proteins are in turn noncovalently bound to the peptidoglycan cell wall via C-terminal surface-layer homology (SLH) repeats [64].

During growth on cellulose, the cellulosome is attached to the cell in early exponential phase, released during late exponential phase, and is found attached to cellulose during stationary phase [64]. Cellulosome expression has been shown to be negatively regulated by cellobiose via a carbon catabolite repression mechanism [28,31], and positively regulated through binding of cellulose and associated polysaccharides to anti-σ factors, allowing cellulosome expression using alternative σ factors [33,34], suggesting that the cellulosome should not be expressed in cellobiose-grown cultures. The ability of *C. thermocellum* to control scaffoldin and cellulase mRNA [25-28] and protein [29-32] levels in response to substrate type and growth rate has been extensively studied, and reveals that expression of cellulosomal enzymes is present in the absence of cellulose, albeit at lower levels. We detected expression of 7 cellulosomal structural proteins, 31 cellulosome-associated glycosidases, and 19 non-cellulosomal CAZymes on cellobiose using 2D-HPLC-MS/MS ( Additional file 3).

Of the 8 encoded non-catalytic cellulosomal proteins, 7 were detected using the combined acquisition methods (shotgun and 4-plex). SdbA (Cthe_1307) was the most abundant anchoring protein, and scaffoldin CipA (Cthe_3077) was found in the top 50% of total proteins detected (RAI = 0.42). OlpB, Orf2p, and OlpA located downstream of CipA (Cthe_3078-3080) were also detected, but at sequentially lower levels. Expression of cellulosomal anchoring proteins Cthe_0452 and Cthe0736 was also detected, but only during 4-plex acquisition. Microarray studies revealed that transcription of *sdbA* was low compared to *cipA, olpB, orf2p*, and *olpA* on cellulose [37], while nano-LC-ESI-MS revealed that SdbA was only expressed in cellobiose-grown cultures [29]. This coincided with our high SdbA levels detected in cellobiose-grown cell-free extracts. On cellulose, Raman *et al.* found no change in *cipA* transcription and a 2-fold increase in *orf2p* transcription in stationary phase [37], while Dror *et al.* observed an increase in transcription of *orf2p* as well as *cipA* and *olpB* with decreasing growth rate [26]. Alternatively, Gold *et al.* showed similar expression of Orf2p relative to CipA in both cellobiose and cellulose-grown samples and increased expression of OlpB in cellobiose-grown cultures [29]. We, however, did not observe any statistically relevant changes of cellulosomal proteins on cellobiose during transition into stationary phase.

*C. thermocellum* encodes 73 glycosidases containing a type I dockerin, 65 of which have been detected and characterized at the protein level [37]. 2D-HPLC-MS/MS of exponential phase cell-free extracts detected 31 cellulosomal glycosidases ( Additional file 3), 19 of which were in the top 90^th^ percentile of total proteins detected (RAI > 0.1). In addition to high RAI levels of CelS, a cellulosomal subunit shown to be highly expressed [25,27], XynC, CelA, XynA/U, CelG, and glycosidase Cthe_0821 were also detected in high amounts. Other characterized cellulosomal glycosidases detected included CelB, XynZ, XghA, CelR, CelK, and CelV. Proteomic analysis has shown that exoglucanases CelS and CelK, and endoglucanase CelJ are higher in cellulose versus cellobiose-grown cultures, while hemicellulases (XynZ, XynC, XynA/U, XghA, Cthe_0032) and endoglucanases belonging to family GH5 (CelB, CelG, Cthe_2193) and GH8 (CelA) were more abundant in cellobiose versus cellulose-grown cultures [29]. This agrees with our relative protein abundance profiles exhibiting high xylanase, GH5 family glycosidase, and CelA expression, and lower CelK and CelJ expression in exponential cellobiose-grown cell-free extracts. Interestingly, despite the presence xylanases, sequence homology-based annotation has not revealed the presence of xylose reductase, xylitol dehydrogenase, xylose isomerase, or xylulokinase required for xylose utilization. This suggests that, in the absence of cellulose, *C. thermocellum* may be predisposed to expressing xylanases, which typically degrade hemicellulosomal xylans, exposing buried cellulose fibres.

With the exception of a 2-fold increase in cellulosomal glycosidases Cthe_0821, Cthe_2761, and Cthe_0745, and a 1.6-fold decrease in XynD (Cthe_0625), no other statistically significant changes were observed in detected cellulosomal cellulases during transition from exponential to stationary phase. While this contradicted high variability in transcription of cellulosomal glycosidases of cellulose-grown cells [37], lack of variability in our experiment may have been attributed to differences in growth substrate used. In fact, Dror *et al.* found negligible changes in transcription of *celB*, *celG*, *celD*, and *celF* between exponential and stationary phase cellobiose-grown cultures [27]. Alternatively, our processing method, which included several wash steps prior to lysing the cells, may have imposed bias and variability by potentially washing off weakly bound cellulosomal glycosidases.

**Figure 2:**
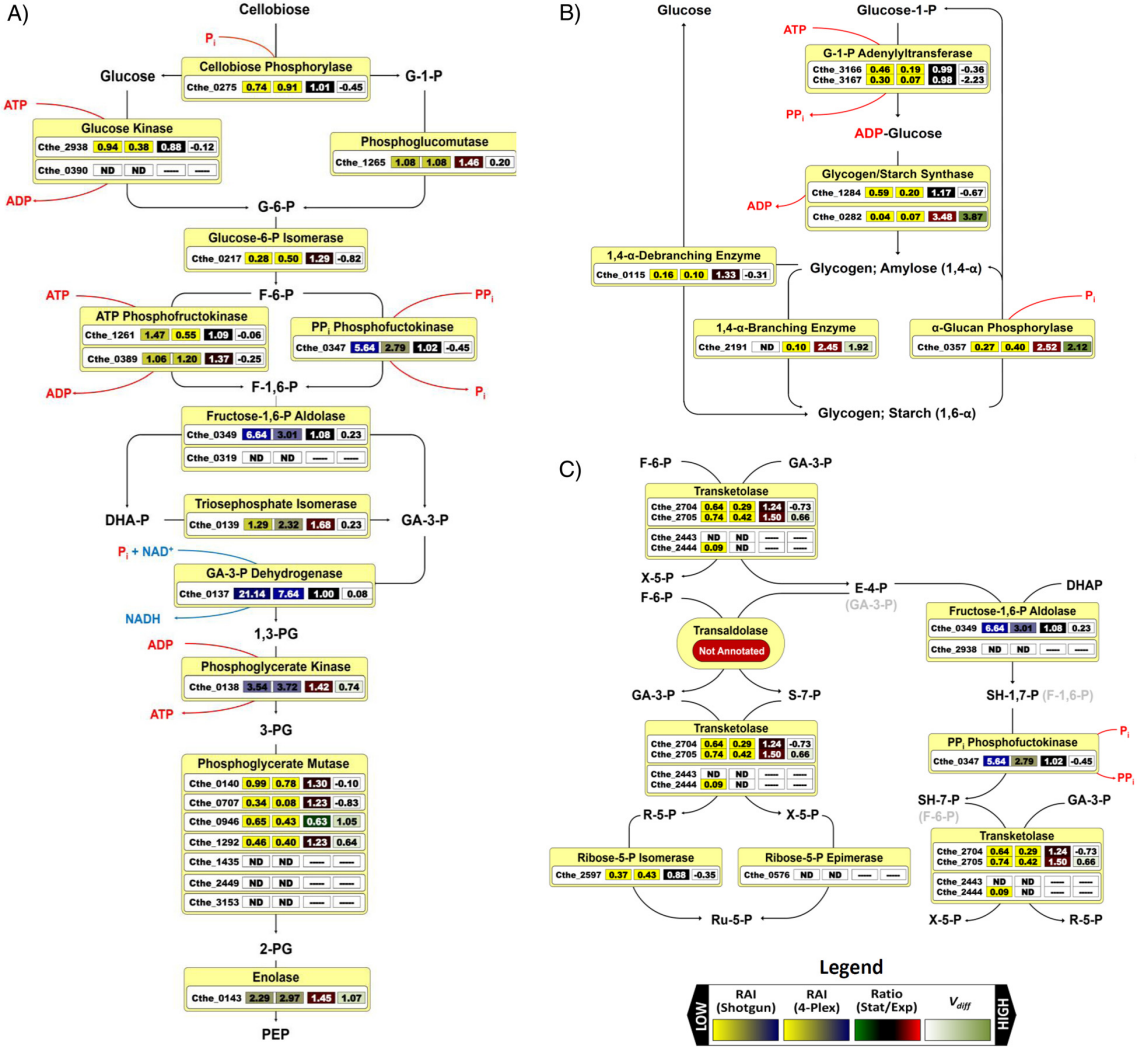
** Relative abundance indexes and changes in protein expression levels of protein involved in glycolysis, glycogen metabolism, and pentose phosphate pathway.** Relative abundance indexes (values 1 and 2), changes in protein expression ratios (value 3), and associated *V*_*diff*_ values (value 4) indicating confidence levels of changes in expression ratios are indicated for enzymes involved in (**A**) glycolysis, (**B**) glycogen metabolism, and (**C**) pentose phosphate pathway. Given the absence of genes encoding transaldolase, we propose an alternative pathway for production of xylulose-5-phosphate and ribose-5-phosphate using fructose-1,6-P aldolase and PP_i_ phosphofructokinase. Metabolites shown in grey are those commonly metabolized by these enzymes. G-1-P, glucose-1-phosphate; G-6-P, glucose-6-phosphate; F-1-P, fructose-1-phosphate; F-1,6-P, fructose-1,6-bisphosphate; DHA-P, dihydroxyacetone phosphate; GA-3-P, glyceraldehydes-3-phosphate; PG, phosphoglycerate; PEP, phosphoenolpyruvate; X-5-P, xylulose-5-phosphate; E-4-P, erythrose-4-phosphate; S-7-P, sedoheptulose-7-phosphate; S-1,7-P, sedoheptulose-1,7-phosphate; R-5-P, ribose-5-phosphate; Ru-5-P, ribulose-5-phosphate.

In addition to cellulosomal glycosidases, 35 non-cellulosomal CAZymes that do not have a dockerin domain are encoded in the genome. Of the 19 non-cellulosomal CAZymes detected in exponential phase cell-free extracts using 2D-HPLC-MS/MS, half had RAI ratios in the top 90% (RAI > 0.1) of total peptides detected. Not surprisingly, the most abundant CAZyme cellobiose phosphorylase Cthe_0275 (glycosyltransferase family 36), which is involved in intracellular phosphorylytic cleavage of cellobiose, fell within the top 25% of detected proteins. Cellobiose phosphorylase Cthe_2989 was also found in high amounts (RAI = 0.23), whereas glycosyltransferase Cthe_1221, a putative cyclic β-1,2 glucan synthetase, was detected in the bottom 10% of all proteins detected (Figure 2a). CelI, an endo-1,4-β-glucanase (Cthe_0040) was not detected, consistent with growth on cellobiose. Other highly abundant non-cellulosomal CAZymes include amidohydrolase (Cthe_1777), glucoamylase (Cthe_1787), xylanase A precursor (Cthe_1911), α-N-arabinofuranosidase (Cthe_2548), CelC (Cthe_2807), and several less characterized glycosidases (Cthe_3163, Cthe_1911, Cthe_2989). While Raman *et al.* report decreased transcription of glycosyltransferases involved in intracellular phosphorolytic cleavage of cellodextrin and cellobiose (family 36), and increased transcription of a number of other CAZymes in response to decreased substrate availability in stationary phase [37], we saw no statistically significant changes in CAZyme expression with two exceptions: LicA (Cthe_2809) increased in stationary phase, consistent with reports by Newcomb and Wu [66] and Raman *et al.*[37], and acetyl xylan esterase (Cthe_3063) also increased contradicting previously reported microarray data [37]. CelC expression (Cthe_2807), which is negatively regulated by the co-transcribed LacI family transcriptional regulator GlyR3 (Cthe_2808), has consistently been shown to increase in the presence of laminaribiose [67] and in stationary phase on cellulose [37] and cellobiose [28]. While CelC expression was shown to have an overall increase in stationary phase among biological replicates, deviation between replicates makes it difficult to tell if this is simply an articat. Finally, of the 7 membrane-associated RsgI-like anti-σ^I^ factors proposed to activate expression of different glycosidases in the presence of cellulose and other polysaccharides, three have been detected (Cthe_0059, Cthe_0267, and Cthe_2521). The binding of a particular polysaccharide to corresponding anti-σ^I^ factor N-terminal carbohydrate binding domains is proposed to promote the C-terminal release of putative alternative σ^I^-factors (SigI) encoded upstream of these anti-σ^I^ factors, allowing for expression of select glycosidases, some of which (ex. CelA) are encoded downstream of the anti-σ^I^ factors that regulate their expression [33,36]. 

#### Cellodextrin transport

Oligosaccharides derived from cellulose hydrolysis are actively transported via ATP-dependent cello-oligosaccharide ABC transporters [68]. Of the five encoded cello-oligosaccharide ABC transporters, only Cthe_0391-0393, Cthe_1018-1020, and Cthe_1862 were detected in significant amounts, consistent with mRNA expression levels reported by Raman *et al.*[37]. While the RAI was low for membrane spanning domains of these transporters, cytoplasmic nucleotide binding domains and extracellular carbohydrate-binding domains (Cbp) had higher RAI values (Figure 2a, Additional file 3). Characterization of Cbp subunits revealed that CbpA (Cthe_0393) binds only to cellotriose, CbpB (Cthe_1020) binds to cellodextrins of different lengths (G2-G5), while CbpC and CbpD (Cthe_2128 and Cthe_2446, respectively) preferentially bind to G3-G5 cellodextrins [34]. Given the absence of cellodextrins longer than cellobiose (G2) in our growth medium, the absence of the latter transporters Cthe_2125-2128 and Cthe_2446-2449 is not surprising. While high expression levels of cellotriose ABC transporter were a bitsurprising given the cells were grown on cellobiose, studies have shown that *C. thermocellum* and other cellulolytic bacteria (ie. *Fibrobacter succinogenes*) are capable of producing cellotriose during growth on cellobiose via reversible cellodextrin phosphorylases [69,70]. While the 2.8-fold increase in Cthe_1020 expression and 2.6-fold decrease in Cthe_0391 expression in stationary phase was statistically significant (*V*_*diff*_ > 1), the other subunits of these transporters did not follow suit.

### Conversion of cellobiose to end-products

#### Glycolysis

In *C. thermocellum*, conversion of glucose to phosphoenolpyruvate (PEP) occurs via the Embden-Meyerhoff-Parnas pathway (Figure 2a, Additional file 4). All glycolytic proteins were detected in the top 20% (RAI > 0.83) of total proteins detected by 2D-HPLC-MS/MS, with a few exceptions. Glucose-6-P isomerase (Cthe_0217) had a RAI = 0.28, and one of the two encoded glucose kinases (Cthe_0390) was not detected. While glyceraldehyde-3-P dehydrogenase was the most highly expressed protein (RAI = 21.1) of all proteins detected, expression of subsequent proteins encoded in the predicted operon (Cthe_0137-0140) decreased respectively with increasing gene distance from glyceraldehyde-3-P dehydrogenase, suggesting transcriptional and/or post-transcriptional regulation of the operon. Protein expression profiles show that interconversion of fructose-1-P to fructose-1,6-bisphosphate can occur via pyrophosphate (PP_i_)-dependent 6-P-fructokinase (RAI = 5.64), which was detected at higher levels than ATP-dependent 6-P-fructokinases Cthe_1261 and Cthe_0389 (RAI = 1.47 and 1.06, respectively). Of the two encoded fructose-1,6-P aldolases (Cthe_0349 and Cthe_2938), only Cthe_0349 was detected. While seven copies of putative phosphoglycerate mutase are encoded, Cthe_0140, which is encoded in a predicted operon containing glyceraldehydes-3-P dehydrogenase, phosphoglycerate kinase, and triosephosphate isomerase (Cthe_0137-0139) shows maximal expression throughout fermentation, consistent with mRNA expression profiles on cellulose [37]. Expression of phosphoglycerate mutase Cthe_0946, Cthe_1292, and Cthe_0707 were also detected, albeit at lower levels than Cthe_0140, while Cthe_1435, Cthe_2449, and Cthe_3153 were not detected.

While the majority of glycolytic proteins did not change during transition to stationary phase, phosphoglycerate kinase and enolase increased by ~1.4-fold with a *V*_*diff*_ confidence score of >0.7, while phosphoglycerate mutase and triosephosphate isomerase increased by ~1.4-fold, but only with a *V*_*diff*_ confidence score of >0.2. While Raman *et al.* (2011) observed a decrease in mRNA expression of ATP-dependent phosphofuctokinase Cthe_1261 and PP_i_-dependent phosphofructokinase Cthe_0389 during transition to stationary phase, we did not observe any changes in protein levels. However, we did observe a decrease in phosphoglycerate mutase Cthe_0946 and an increase in Cthe_1292, consistent with cellulose grown *C. thermocellum* mRNA profiles [37].

#### Energy storage

Glycogen, an energy and carbon storage compound, is commonly synthesized during periods of slow or no growth, especially in carbon excess, and is often associated with sporulation [71,72]. Glucose-1-P adenylyltransferase (Cthe_3166 and Cthe_3167), involved in the synthesis of the primary glucosyl donor ADP-glucose, was detected in exponential phase cell-free extracts using shotgun 2D-HPLC-MS/MS (Figure 2b, Additional file 4). Of the two genes encoding glycogen synthase (Cthe_1284 and Cthe_0282), which catalyzes α-1,4-glucosyl linkages to a pre-existing α-1,4-glucan, levels of Cthe_1284 were ~15-fold higher than that of Cthe_0282, suggesting it is the primary glycogen synthase in *C. thermocellum*. While the level of 1,4-α-glucan branching enzyme, required for catalyzing α-1,6-glucosyl linkages, was below our threshold cutoff in shotgun analysis, it was detected in 4-plex analysis. A putative 1,4-α-glycogen debranching enzyme and α-glucan phosphorylase, required for glycogen breakdown, was also detected in exponential phase cultures. On the basis of simultaneous glucose-1-P adenylyltransferase, glycogen synthase, and glycogen phosphorylase activities in *C. cellulolyticum* cell-free extracts, Guedon *et al.* have proposed that glycogen synthesis and glycogenolysis can occur simultaneously [73]. While allosteric regulation of these enzymes has been demonstrated in *E. coli*[71], the effect of allosteric regulators on these enzymes was not studied in *C. cellulolyticum*. Alternatively, the simultaneous detection of enzymes involved in glycogen synthesis as well as glycogen breakdown may be a consequence of metabolic heterogeneity within the culture, where some cells are expressing pathways for glycogen synthesis while others are expression pathways capable of glycogenolysis. While this type of cell-to-cell variation has been observed in *Bacillus subtilis*[74], it cannot be verified using proteomics as these variations are homogenized as one examines bulk mixtures of cells.

We observed a 3.5-fold increase in glycogen synthase Cthe_0282 and a 2.5-fold increase in 1,4-α-branching enzyme in stationary phase, suggesting that glycogen synthesis is favoured during stationary phase. While glucose-1-P adenylyltransferase expression did not change, its activity has been shown to allosterically activated via glycolytic intermediates and inhibited via AMP, ADP, P_i_, and PP_i_[71,72]. While enzyme assays show that levels of glucose-1-P adenelylytransferase and glycogen synthase increase with decreasing growth rate during transition to stationary phase in most organisms [71], catalytic activities of these enzymes, as well as α-glucan phosphorylase activity, increased with higher growth rates in *C. cellulolyticum*[73]. Furthermore, in contrast to many bacterial species, which produce glycogen during the onset of stationary phase, glycogen synthesis reached a maximum in exponential phase and was utilized during transition to stationary phase in batch *C. cellulolyticum* cultures [73]. Interestingly, expression of α-glucan phosphorylase also increased 2.5-fold, which may help the cell utilize glycogen in the absence of an external carbon source.

#### Pentose phosphate pathway

The oxidative branch of the pentose phosphate pathway (PPP) generates reducing equivalents (NADPH) for biosynthesis, whereas the non-oxidative branch produces key intermediates, namely ribose-5-P and erythrose-4-P, required for the synthesis of nucleotides and aromatic amino acids, respectively. The absence of genes encoding glucose-6-P dehydrogenase, gluconolactonase, and 6-P-gluconate dehydrogenase of the oxidative PPP branch suggests that an alternative NADPH generation system must exist and that glycolytic intermediates (glyceraldehydes-3-phosphate and fructose-6-phosphate) must feed the non-oxidative branch of the PPP (Figure 2c. Additional file 4). Furthermore, homology-based annotation suggests that the non-oxidative branch of the PPP is incomplete. While *C. thermocellum* encodes ribulose-5-P isomerase, ribulose-5-P epimerase, and two transketolases (Cthe_2443-2444 and Cthe_2704-2705), no gene encoding a transaldolase has been identified. 2D-HPLC-MS/MS expression profiles reveal that transketolase Cthe_2704-2705 is highly expressed throughout fermentation (RAI ~ 0.7), while Cthe_2443 is not detected and Cthe_2444 is found only in low amounts (RAI = 0.09). While ribose-5-P isomerase was detected (RAI = 0.37), ribose-5-P epimerase was not. Given the absence of transaldolase, ribose-5-phosphate must be synthesized using an alternative pathway.

A novel mechanism of non-oxidative hexose-to-pentose conversion that does not require transaldolase has been demonstrated in *Entamoeba histolytica* and other parasitic protists [75-77]. This system employs transketolase, aldolase, and PP_i_-dependent 6-phosphofructokinase (Figure 2c). Susskind *et al.* have shown that fructose-1,6-bisphosphate aldolase, which typically converts glyceraldehyde-3-P and dihydroxyacetone-P into fructose-1,6-bisphosphate, is capable of converting dihydroxyacetone-P and erythrose-4-P into sedoheptulose-1,7-bisphosphate [77]. PP_i_-dependent phosphofructokinase, which commonly catalyzes the reversible interconversion of fructose-6-P to fructose-1,6-bisphosphate, can then produce sedoheptulose-7-bisphosphate from sedoheptulose-1,7-bisphosphate. Finally sedoheptulose-7-bisphosphate and glyceraldehydes-3-P can be converted to ribose-5-P and xylose-5-P using transketolase again. While enzyme assays have not been carried out to determine the substrate specificity of fructose-1,6-bisphosphate aldolase and PP_i_-dependent 6-phosphofructokinase in *C. thermocellum*, it is tempting to propose a similar hexose-to-pentose conversion mechanism.

#### Pyruvate formation from phosphoenolpyruvate

**Figure 3:**
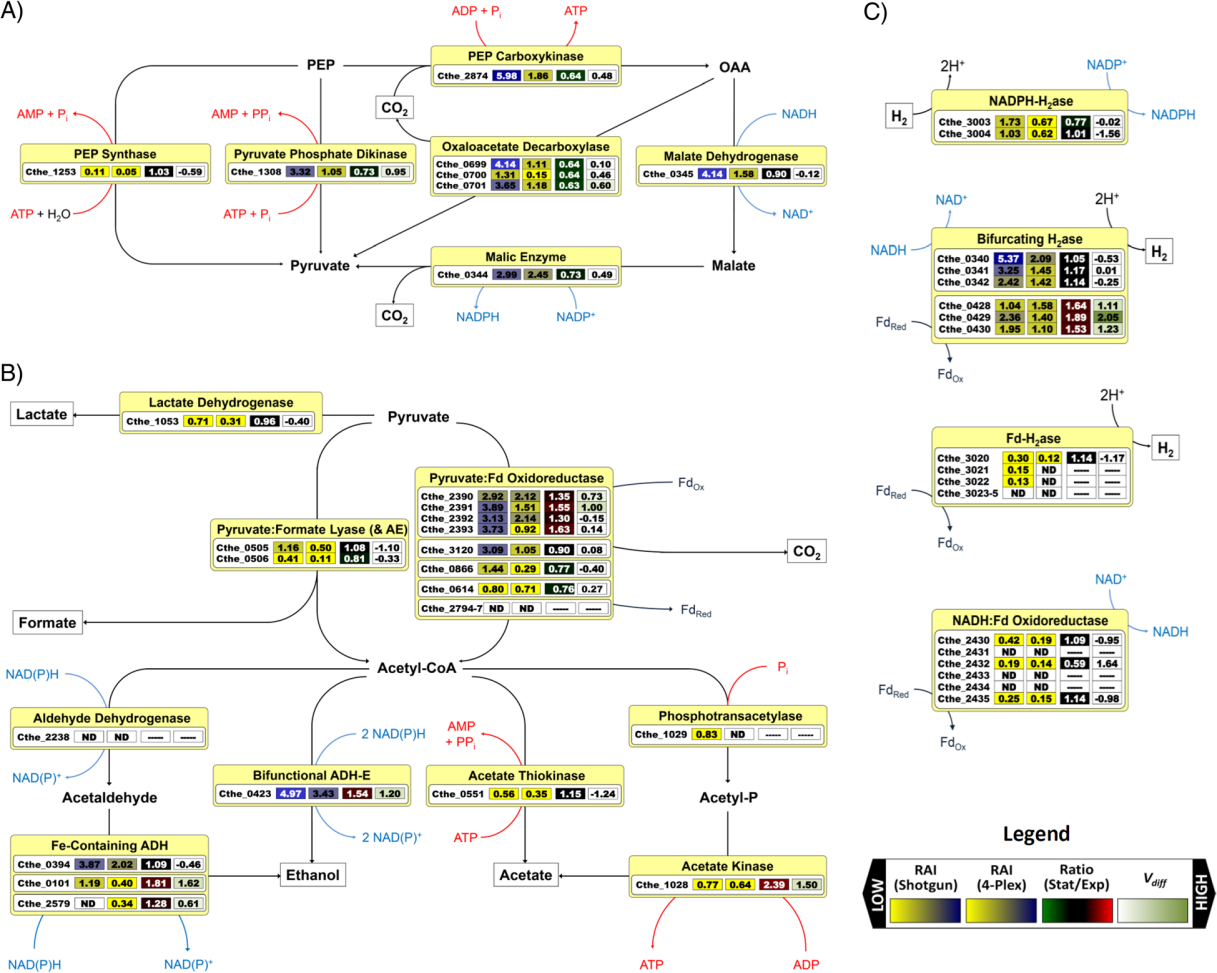
** Relative abundance indexes and changes in protein expression levels of proteins involved in conversion of phosphoenolpyruvate to end-products.** Relative abundance indexes (values 1 and 2), changes in protein expression ratios (value 3), and associated *V*_*diff*_ values (value 4) indicating confidence levels of changes in expression ratios for enzymes involved in (**A**) conversion of phosphoenolpyruvate to pyruvate (**B**) catabolism of pyruvate into end-products, and (**C**) electron transfer pathways between ferredoxin (Fd), NAD-(P)H, and H_2_. PEP, phosphoenol pyruvate; OAA, oxaloacetate; Fd, ferredoxin.

While most organisms convert phosphoenolpyruvate (PEP) to pyruvate via pyruvate kinase, producing ATP from ADP [78], sequence homology-based annotation has not revealed the presence of a pyruvate kinase in *C. thermocellum*. However, several alternative proteins are expressed that may result in a tightly regulated pathway node (Figure 3a, Additional file 4) leading to pyruvate synthesis. Phosphoenolpyruvate can be reversibly converted to pyruvate via pyruvate phosphate dikinase (PPDK), producing ATP and P_i_ from AMP, and PP_i_, or using PEP synthase (PEPS) which produces ATP and H_2_O from AMP, and P_i_. While PPDK was expressed at high levels in exponential phase, PEPS was not (RAI = 3.32 *vs* 0.11). Alternatively, PEP carboxykinase (PEPCK), which was also highly expressed (RAI = 5.98), can convert PEP to oxaloacetate while generating ATP. Oxaloacetate can subsequently be converted either directly to pyruvate via oxaloacetate decarboxylase (OAADC), or indirectly through malate via malate dehydrogenase (MDH) and malic enzyme (ME), all of which were also highly expressed. High NADH-dependent MDH and NADP^+^-dependent ME activities (Rydzak *et al.*, *unpublished*) suggest that MDH/ME facilitate transhydrogenation from NADH to NADP^+^, resulting in NADPH for biosynthesis, or potential H_2_ or ethanol synthesis [55]. Interestingly, all the enzymes in this node, with the exception of PEPS and MDH, decrease ~1.4 to 1.6-fold during stationary phase, generally consistent with reported mRNA profiles of cellulose grown cells [37]. Regulation of carbon flux through this node cannot be simply attributed to changes in protein expression level since ME has been shown to be regulated allosterically. Ammonia has been reported as an activator of ME in *C. thermocellum*, and thus, transhydrogenation of NADH to NADP^+^ via MDH and ME is only allowed when sufficient NH_4_^+^ is present for biosynthesis [79]. More recently, PP_i_ inhibition of ME has been demonstrated (Taillefer and Sparling, *unpublished*). While this may be counterintuitive given that high levels of PP_i_ are present in the cell during rapid growth and biosynthesis, which in turn increases the demand for NADPH, the regulatory aspects with MDH and ME are tightly knit with PPDK, which either uses PP_i_ during glycolysis, allowing for NADPH formation using MDH and ME, or produces PP_i_ during carbon starvation and gluconeogenesis, inhibiting the MDH/ME pathway accordingly to the cells NADPH demand. 

#### Pyruvate Catabolism and end-product synthesis

Synthesis of organic end-products from pyruvate is mediated by enzymes comprising two major branchpoints, namely the *pyruvate/acetyl-CoA/lactate* branchpoint and the *acetyl-CoA/ethanol/acetate* branchpoint, while H_2_ can be generated from reduced ferredoxin (Fd_r_), NADH, or NADPH using multiple hydrogenase (H_2_ase) complexes (Figure 3). While the functionality of these pathways has been verified using enzyme assays [4,55], and transcriptional expression of the genes involved in these pathways has recently been elucidated [22,36,37], there have been no reports regarding the expression levels of these genes at the protein level. Given that there are apparent redundancies in genes encoding enzymes with analogous functions (e.g. pyruvate:ferredoxin oxidoreductases, alcohol dehydrogenases, hydrogenases) according to the current annotation, it is important that protein abundances and their expression profiles under physiological conditions be determined for the effective application of metabolic engineering strategies to improve rates and/or yields of H_2_, ethanol, and other desired end-products.

#### Pyruvate/acetyl-CoA/lactate branchpoint

*C. thermocellum* may convert pyruvate into (i) CO_2_, Fd_r_, and acetyl-CoA, (ii) formate and acetyl-CoA, and (iii) lactate via pyruvate:ferredoxin oxidoreductase (POR), pyruvate:formate lyase (PFL), and lactate dehydrogenase (LDH), respectively [4]. Based on end-product profiles (Figure 1), carbon flux is preferentially channelled through POR. *C. thermocellum* encodes two 4-subunit PORs. While the γ, δ, α, and β subunits encoded by the gene cluster Cthe_2390-2393 are highly expressed, proteins encoded by Cthe_2794-2797 are not detected by 2D-HPLC-MS/MS, in agreement with mRNA profiles reported by Raman *et al.*[37] and Fong *et al.*[80]. This contrasted with RT-PCR experiments performed by Carere *et al.*, who reported high expression of subunit Cthe_2796 and low expression of subunit Cthe_2392 in exponential phase cultures grown on cellulose [22].

Three putative single subunit POR-like oxidoreductases, including Cthe_3120, a putative pyruvate:flavodoxin oixidoreductase, Cthe_0866, a putative 2-oxogluterate synthase, and Cthe_0614, a putative indolepyruvate:fd oxidoreuctase, were also detected at high levels using 2D-HPLC-MS/MS. In agreement with our relative protein abundance profiles, RT-PCR experiments have confirmed high expression levels of Cthe_3120 [22]. Given that BLAST analysis of Cthe_3120 revealed homology to a characterized pyruvate-dependent POR isolated from *C. acetobutylicum*[81], also found in a number of *Thermoanaerobacter* species, these oxidoreductases may also be capable of converting pyruvate into acetyl-CoA.

Formate production was consistent with the presence of PFL (Cthe_0505). While a number of studies have reported formate production [3-5,35,55], others have not [50,68,82]. These discrepancies may be a result of the use of different detection methods (gas chromatography *vs* high pressure liquid chromatography), fermentation conditions (batch with no pH control *vs* bioreactor with pH control), or media composition (complex *vs* minimal). Expression levels of PFL were lower than that of POR Cthe_2390-2393, in agreement with end-product accumulation rates and previously reported enzyme activities [4]. Of the four putative PFL-activating enzymes (Cthe_0506, Cthe_0647, Cthe_1167, Cthe_1578) required for glycyl radical formation on the C-terminal portion of PFL [83,84], only Cthe_0506 was detected. While this agreed with high mRNA levels in cellobiose [22] and cellulose grown batch cultures [37], Raman *et al.* also reported high expression levels of Cthe_0647 during fermentation. While PFL and PFL-activating enzyme Cthe_0506 are encoded next to each other, the 3-fold difference in expression levels suggests that they are either transcribed independently as in *Streptococcus bovis*[85], or have different protein stabilities.

While LDH was expressed, albeit at lower levels than detected PORs and PFL, lactate production was not detected under the conditions tested. In *C. thermocellum* LDH has been shown to be allosterically activated by fructose-1,6-bisphosphate (FDP), [20] while in *Caldicellulosiruptor saccharolyticus*, a close relative to *C. thermocellum*, LDH is activated by FDP and ATP, and inhibited by NAD^+^ and PP_i_[21]. While lactate production in *C. thermocellum* was observed in batch cultures under carbon excess [3] and low culture pH (Rydzak *et al. unpublished*), this may be due to high intracellular FDP, concentrations, high NADH/NAD^+^ ratios, and/or high ATP/PP_i_ ratios during transition to stationary phase [21], which may have not been reached under our growth conditions.

#### Acetyl-CoA/ethanol/acetate branchpoint

Catabolism of acetyl-CoA into ethanol and acetate plays an important role in NADH reoxidation and energy conservation, respectively. Acetyl-CoA can be converted into ethanol directly using a bi-functional acetaldehyde/alcohol dehydrogenase (Cthe_0423; *adhE*), or indirectly via an NADH-dependant aldehyde dehydrogenase (Cthe_2238; *aldH*) and a number of iron containing alcohol dehydrogenases (Cthe_0101, Cthe_0394, Cthe_2579; *adh*). Expression of Cthe_2238 (*aldH*), Cthe_0394 (*adhY*), and Cthe_2579 (*adhZ*) has been confirmed by real-time PCR [35]. Of these ADHs, AdhE was the most abundant ADH detected (Figure 3b). Low expression levels of AldH suggest that AdhE is the primary protein involved in acetyl-CoA reduction and ethanol formation. ADHs Cthe_0394, Cthe_0101, and Cthe_2579 were expressed at 78%, 24%, and 9% of the levels of AdhE, respectively, suggesting that they may also be involved in formation of ethanol from acetaldehyde, albeit at lower levels. Two other zinc-containing ADH GroES-like heat shock proteins, Cthe_0388 and Cthe_2445, were also detected, the former being more highly expressed ( Additional file 4). While crude cell-free extract enzyme activities have shown the presence of both NADH and NADPH-dependent ADH activities, sequence analysis could not verify the substrate specificities of these enzymes.

Acetyl-CoA can be converted into acetate directly via acetate thiokinase (ATK) or indirectly through an acetyl phosphate intermediate using contiguously encoded phosphotransacetylase (PTA) and acetate kinase (ACK). While activities of PTA (Cthe_1029) and ACK (Cthe_1028) have been verified in *C. thermocellum*[50], and ACK has been purified and characterized [86], the substrate specificity of the putative ATK (Cthe_0551) has not been determined. Although both reactions generate ATP, ATK does so using AMP and PP_i_, whereas PTA and ACK use ADP and P_i_. This in turn has an impact on the thermodynamics of each reaction. The free energy of acetate production using PTA and ACK is more thermodynamically favourable than using ATK (ΔG˚’ = −4 kJ mol^-1^*vs* +9 kJ mol^-1^), and thus PTA and ACK are proposed to favour acetate production from acetyl-CoA, while ATK favours acetyl-CoA production from acetate. While Raman *et al.* report low mRNA levels of *pta* and *ack* and higher levels of *atk*[37], 2D-HPLC-MS/MS showed that all three proteins were detected at comparable levels (Figure 3b). Expression of all three enzymes remained constant throughout fermentation.

#### H_2_ generation pathways

The genome of *C. thermocellum* encodes four putative hydrogenases (H_2_ases), including an energy conserving Ech-like Fd-dependent [NiFe]-H_2_ase (Cthe_3019-3024) and 3 Fe-only H_2_ase catalytic subunits (Cthe_0342, Cthe_0430, Cthe_3003). Transcription of all of these subunits has been confirmed using RT-PCR [22]. Enzyme assays have shown that NADPH-dependent H_2_ase activity is 5 to 10-fold higher than Fd and NADH-dependant H_2_ase activities [4,55]. The presence of a gene similar to the NADPH-binding subunit of glutamate synthase (Cthe_3004) adjacent to Cthe_3003 suggests that it may form a dimer with Cthe_3003 capable of generating NADPH from H_2_[18]. 2D-HPLC-MS/MS reveals that both subunits are highly expressed, while subunits comprising both Fd-dependent [NiFe]-H_2_ase and *Rhodobacter* nitrogen fixation (RNF)-like NADH:Fd oxidoreductase were detected in low amounts or not at all (Figure 3c), consistent with enzyme activity profiles [4,55] and mRNA profiles [37].

This leads to the question of how reduced Fd, formed by PFO, is reoxidized. Recently a heterotrimeric bifurcating H_2_ase, which utilize reduced Fd and NADH synergistically to overcome the thermodynamic barrier of NADH oxidation for H_2_ production, has been purified and characterized in *Thermotoga maritima*[87]. Genomic organization of Fe-only H_2_ases Cthe_0342 and Cthe_0430 suggests that they may form bifurcating heterotrimers with neighbouring Nuo-like gene products Cthe_0340/0341 and Cthe_0428/0429, respectively. Both Cthe_0340-0342 and Cthe_0428-0430 were detected in high amounts, providing a probable method for Fd reoxidation. These putatively bifurcating H_2_ases may be responsible for the low NADH-dependent H_2_ase activities detected in cell-free extracts. While these activities may be higher in the presence of reduced Fd, bifurcating H_2_ase activities could not be assayed in cell-free extracts, and thus purification of these H_2_ases is required for validation of bifurcating activity.

Interestingly, genomic organization of *C. thermocellum* H_2_ase subunits and upstream regulatory elements (see below) of Cthe_0428-0430, Cthe_0340-0342, and Cthe_3019-3014 reveal high similarity to that of *Thermoanaerobacterum saccharolyticus* (a.k.a. *T. thermosaccharolyticus*) gene clusters *hfs*, *hyd*, *ech*, respectively. While all three H_2_ases were expressed in wild-type *T. saccharolyticus*, as demonstrated by real-time PCR, gene knockout studies revealed that: i) *hfs* was the primary H_2_ase responsible for H_2_ production as its deletion drastically decreased H_2_ production; ii) *hyd* knockouts had no effect on H_2_ yields in batch fermentations, but decreased total methyl viologen-dependent H_2_ase activity compared to wild type cells; and iii) *ech* knockouts had no effect on H_2_ production or methyl viologen-dependent H_2_ase activity [88]. This demonstrates the importance of mutational studies to determine the physiological role of H_2_ases.

#### Changes in expression of enzymes involved in pyruvate catabolism and end-product synthesis

The subtle decrease in formate production rate and inversion of acetate-to-ethanol ratio during transition from exponential to stationary phase are consistent with previous studies [37]. Transition from early to late log phase in pH regulated batch cultures [89], decreasing pH in steady state continuous cultures [90], and increasing dilution rates [73] have all resulted in a shift from acetate to lactate and/or ethanol production mediated by an increase in NADH/NAD^+^ ratios in *C. cellulolyticum*. Similarly, pH controlled batch cultures of *Caldicellulosiruptor saccharolyticus* exhibited increased NADH/NAD^+^ ratios as cells approached mid to late-log phase, which subsequently triggered lactate production thus rebalancing NADH/NAD^+^ ratios in late log and stationary phase [21]. These changes were also accompanied by an increase in LDH and ADH activity, despite the absence of ethanol production. While these studies were performed under carbon excess conditions resulting in prolonged growth and more pronounced changes in end-product ratios, parallels can be drawn with our carbon limited *C. thermocellum* studies.

The ~1.4-fold increase in POR Cthe_2390-2393 and constant expression of PFL during stationary phase (Figure 3c) may divert carbon and electron flux away from PFL explaining the decrease in formate production rates as cells enter stationary phase. The additional reduced Fd produced via PFO must then be reoxidized using Fd-dependant or bifurcating H_2_ases. Accordingly, expression of bifurcating H_2_ase Cthe_0428-0430 increases >1.5-fold in stationary phase. While both bifurcating H_2_ases (Cthe_0428-0430 and Cthe_0340-342) contain various upstream regulatory elements including phosphatases, kinases, and/or PAS/PAC sensors potentially capable of regulating transcription in response to H_2_ levels or redox changes via a two-component regulatory system as in *Ralstonia eutropha*[17,91,92], only Cthe_0428-0430 expression changed under the conditions tested. Regulation of a NAD(H)-dependent Fe-only H_2_ase containing an upstream histidine and serine/threonine protein kinase has also been reported in *Ta. tencongensis*, in which a fourfold decrease in NAD(H)-dependent H_2_ase activity was accompanied by an increase in AldH and ADH activities in response to high H_2_ partial pressures [19].

Providing that NADH/NAD^+^ ratios increase during transition from exponential to stationary phase as in *C. cellulolyticum* and *Ca. saccharolyticus*, the observed increase in select ADHs [AdhE (Cthe_0423), Cthe_0101, glutamyl reductase (Cthe_1863), and groES (Cthe_0388)] during stationary phase may help *C. thermocellum* reoxidize NADH and concomitantly produce ethanol, which explains the observed inversion of acetate-to-ethanol ratio. A similar mechanism of increasing expression of select ADHs to dispose of reducing equivalents during growth and ethanol accumulation is employed by *Thermoanaerobacter* species [93]. Surprisingly, we observed a 2.4-fold increase in acetate kinase expression in stationary phase despite having lower acetate to ethanol ratios. This differs from the mRNA expression profiles on cellulose reported by Raman *et al.*[37]. However, 4-plex 2D-HPLC-MS/MS did not detect the presence of PTA required for production of acetyl-P, and thus changes in expression profiles of PTA in response to growth phase could not be determined.

#### Energy generation and pyrophosphate (PP_i_) metabolism

In addition to substrate level phosphorylation mediated by 1,3-phosphoglycerate kinase, pyruvate phosphate dikinase, phosphoenolpyruvate carboxykinase, acetate kinase, and acetate thiokinase (see above), ATP can also be generated using ATP synthase powered by a proton motive force (PMF). While two types of ATP synthases were detected, including the F-type (Cthe_2602-2609) and the V-type (Cthe_2262-2269), overall expression of the latter was higher ( Additional file 2). Expression of both ATP synthases was generally consistent throughout growth. Given the low expression of both ech H_2_ase and NADH:Fd oxidoreductase, which are predicted to pump out H^+^ and Na^+^, respectively, across the cell membrane during oxidation of reduced Fd, PMF generation for ATP synthesis using these enzymes seems unlikely. While, PMF may be generated through PP_i_ hydrolysis using a membrane bound proton-translocating pyrophosphatase (PPase), the directionality of this PPase is unknown, and may in fact use PMF for PP_i_ synthesis.

PP_i_ is a by-product of various endergonic biosynthetic reactions, including poly-nucleic acid synthesis from (deoxy)nucleotide triphosphates and activation of amino acids, carbohydrates, and fatty acids for protein, polysaccharide, and lipid synthesis [21]. Thus, the effective removal of PP_i_ improves the thermodynamic feasibility of these reactions. Concentrations as low as 2 mM PP_i_ have shown to inhibit growth of some bacteria [94]. In addition to serving as a central energy carrier, PP_i_ serves to regulate key enzymes in carbohydrate metabolism including LDH in *Ca. saccharolyticus*[21], malic enzyme in *C. thermocellum* (Taillefer and Sparling, *unpublished*), ATP-dependent PFK in *T. maritima*[95], and PTA in *C. acidiurici*[96].

As mentioned above, PP_i_ can be utilized in the glycolytic direction by (i) PP_i_-dependent 6-P-fructokinase, (ii) PPDK, and (iii) acetate thiokinase. Alternatively, hydrolysis of PP_i_ via a membrane-bound PPase (Cthe_1425) can be coupled to PMF generation that could be utilized for transport of nutrients, motility, and ATP synthesis. The PP_i_-dependent enzymes used by *C. thermocellum* have remarkable similarities to that of parasitic protists (ie. *Trichomonas foetus*, *Entamoeba histolytica*; [75]) and other bacteria such as *Ca. saccharolyticus*[97]. PP_i_ levels in *Ca. saccharolyticus* have been shown to be elevated (4 ± 2 mM) during exponential phase and lower during transition to stationary phase [97], consistent with other organisms that do not contain a cystolic PPase (*C. thermoaceticum* and *C. pasteuranum*; [98]). Conversely, PP_i_ levels in *E. coli*, which possesses a cystolic PPase, were low (0.3 mM) and did not fluctuate during growth [98]. We observed a 1.9-fold increase in membrane-bound PPase expression in stationary phase cells.

## Conclusions

A unified understanding of how gene and gene-product expression, stability, and regulation, in conjunction with intracellular metabolic profiling and thermodynamics of product formation, are key elements for targeted metabolic engineering strategies and fermentation optimization for the economic feasibility of biofuels production via consolidated bioprocessing. *Clostridium thermocellum*, like many cellulolytic, fermentative, biofuel producing organisms, has multiple enzymes capable of catalyzing parallel reactions and branched product pathways. Measuring peptide spectral counts via shotgun proteomics has been shown to be a valid method for determining relative protein abundance profiles [57-60]. In turn, understanding protein expression profiles may provide genetic engineering strategies targeted at redirecting carbon and electron flux for the optimization of end-product production. Furthermore, responses of protein expression in response to physiological conditions (ie. change in pH, end-product accumulation, carbon limitation) are essential in optimization of growth parameters during fermentation. In this study we performed proteomic analysis of core metabolic proteins involved in (hemi)cellulose degradation and conversion of cellobiose into end-products in order to determine relative expression profiles of key enzyme dictating these pathways, and their changes in expression during their transition from exponential and stationary phase under closed-batch cellobiose-limited conditions.

Using shotgun 2D-HPLC-MS/MS, we determined relative protein expression profiles based on peptide spectral counts in order to identify which proteins and metabolic networks are likely to be utilized during conversion of cellobiose to end-products. We observed differential expression of proteins with the same putative function as well as those capable of parallel reactions that can interconvert one metabolite into another while using different cofactors. Relative protein abundance profiles suggest that ethanol production occurs primarily via AdhE, while H_2_ production occurs via a putative bifurcating H_2_ase and/or a NADPH-dependent H_2_ase. While the majority of proteins involved in central metabolism did not change during transition from exponential to stationary phase, 4-plex 2D-HPLC-MS/MS on iTRAQ labeled samples revealed a 1.4-fold increase in pyruvate:ferredoxin oxidoreductase (Cthe_2390-2393) and a >1.5-fold increase in putative bifurcating hydrogenase, AdhE (Cthe_0423), and alcohol dehydrogenase (Cthe_0101) in stationary phase cell-free lysates, which reflect a decrease in formate production rates and the slight increase in ethanol to acetate ratios.

While we must further examine the physiological stimuli dictating not only gene and protein expression, but intracellular metabolite levels that may regulate carbon and electron flux via allosteric regulation and thermodynamic efficiencies, we have shown that differential protein expression levels under the conditions tested can influence end-product synthesis. Combined knowledge of relative protein expression levels and their changes in response to physiological conditions may aid in targeted metabolic engineering strategies and optimization of fermentation condition for improvement of biofuels production.

## Authors' contributions

TR, JAW, DBL, OVK, and RS conceived and designed the study. TR performed growth studies, end-product analysis, processed samples for proteomic analysis, analyzed proteomic data, and drafted the manuscript with input from DBL and RS. OVK, PDM, RCD, and DS aided in sample processing for proteomic analysis. PE and OVK performed MS runs. VS performed statistical analysis on MS data. All authors read and approved the final manuscript.

## Supplementary Material

Additional file 1 ** Relative abundance index (RAI) distribution using single-plex and 4-plex 2D-HPLC-MS/MS.** RAI distribution values follow a similar trend using both acquisition methods, however RAI per given protein was lower using 4-plex 2D-HPLC-MS/MS. Click here for file

Additional file 2 ** Correlation of protein iTRAQ ratios for biological replicates.** Protein z-score value ratios (A) among stationary and exponential phase biological replicates (reporter ion ratio 114/115 *vs* 116/117) and (B) between stationary *vs* exponential phase cell-free extracts (reporter ion ratio 116/114 *vs* 117/115) illustrating correlation between biological replicates. Positive correlation is represented by points in quadrants 1 and 3. Click here for file

Additional file 3 ** Relative abundance indexes and changes in protein expression levels of proteins involved in conversion of phosphoenolpyruvate to end-products.** Shotgun and 4-plex 2D-HPLCMS/MS data identifying protein relative abundance indexes, changes in protein expression, and vector differences indicating statistical relevance of changes in expression. Click here for file

Additional file 4 ** Relative abundance indexes and changes in protein expression levels of proteins involved in conversion of phosphoenolpyruvate to end-products.** Shotgun and 4-plex 2D-HPLCMS/MS data identifying protein relative abundance indexes, changes in protein expression, and vector differences indicating statistical relevance of changes in expression. Click here for file
