# Supplementary material for: Proteomic analysis of Clostridium thermocellum core metabolism: relative protein expression profiles and growth phase-dependent changes in protein expression
Source: BMC Microbiol. 2012 Sep 21;12:214. doi: 10.1186/1471-2180-12-214 (PMC3492117; doi:10.1186/1471-2180-12-214)
Supplement: Additional file 1 — Relative abundance index (RAI) distribution using single-plex and 4-plex 2D-HPLC-MS/MS. RAI distribution values follow a similar trend using both acquisition methods, however RAI per given protein was lower using 4-plex 2D-HPLC-MS/MS. [file 1471-2180-12-214-S1.docx]

**Additional file 1: Relative abundance index (RAI) distribution using single-plex and 4-plex 2D-HPLC-MS/MS**. RAI distribution values follow a similar trend using both acquisition methods, however RAI per given protein was lower using 4-plex 2D-HPLC-MS/MS.
