# Supplementary material for: Proteomic analysis of Clostridium thermocellum core metabolism: relative protein expression profiles and growth phase-dependent changes in protein expression
Source: BMC Microbiol. 2012 Sep 21;12:214. doi: 10.1186/1471-2180-12-214 (PMC3492117; doi:10.1186/1471-2180-12-214)
Supplement: Additional file 2 — Correlation of protein iTRAQ ratios for biological replicates. Protein z-score value ratios (A) among stationary and exponential phase biological replicates (reporter ion ratio 114/115 vs 116/117) and (B) between stationary vs exponential phase cell-free extracts (reporter ion ratio 116/114 vs 117/115) illustrating correlation between biological replicates. Positive correlation is represented by points in quadrants 1 and 3. [file 1471-2180-12-214-S2.docx]

**A)**

**B)**

**Additional file 2: Correlation of protein iTRAQ ratios for biological replicates.** Protein z-score value ratios (A) among stationary and exponential phase biological replicates (reporter ion ratio 114/115 *vs* 116/117) and (B) between stationary *vs* exponential phase cell lysates (reporter ion ratio 116/114 *vs* 117/115) illustrating correlation between biological replicates. Positive correlation is represented by points in quadrants 1 and 3.
